# Supplementary material for: Pyrotinib Targeted EGFR-STAT3/CD24 Loop-Mediated Cell Viability in TSC
Source: Cells. 2022 Sep 29;11(19):3064. doi: 10.3390/cells11193064 (PMC9563285; doi:10.3390/cells11193064)
Supplement: Supplementary file 1 [file cells-11-03064-s001.zip › cells-1888192-supplementary.pdf]

*Supplementary file*

# Pyrotinib Targeted EGFR-STAT3/CD24 Loop-Mediated Cell Viability in TSC

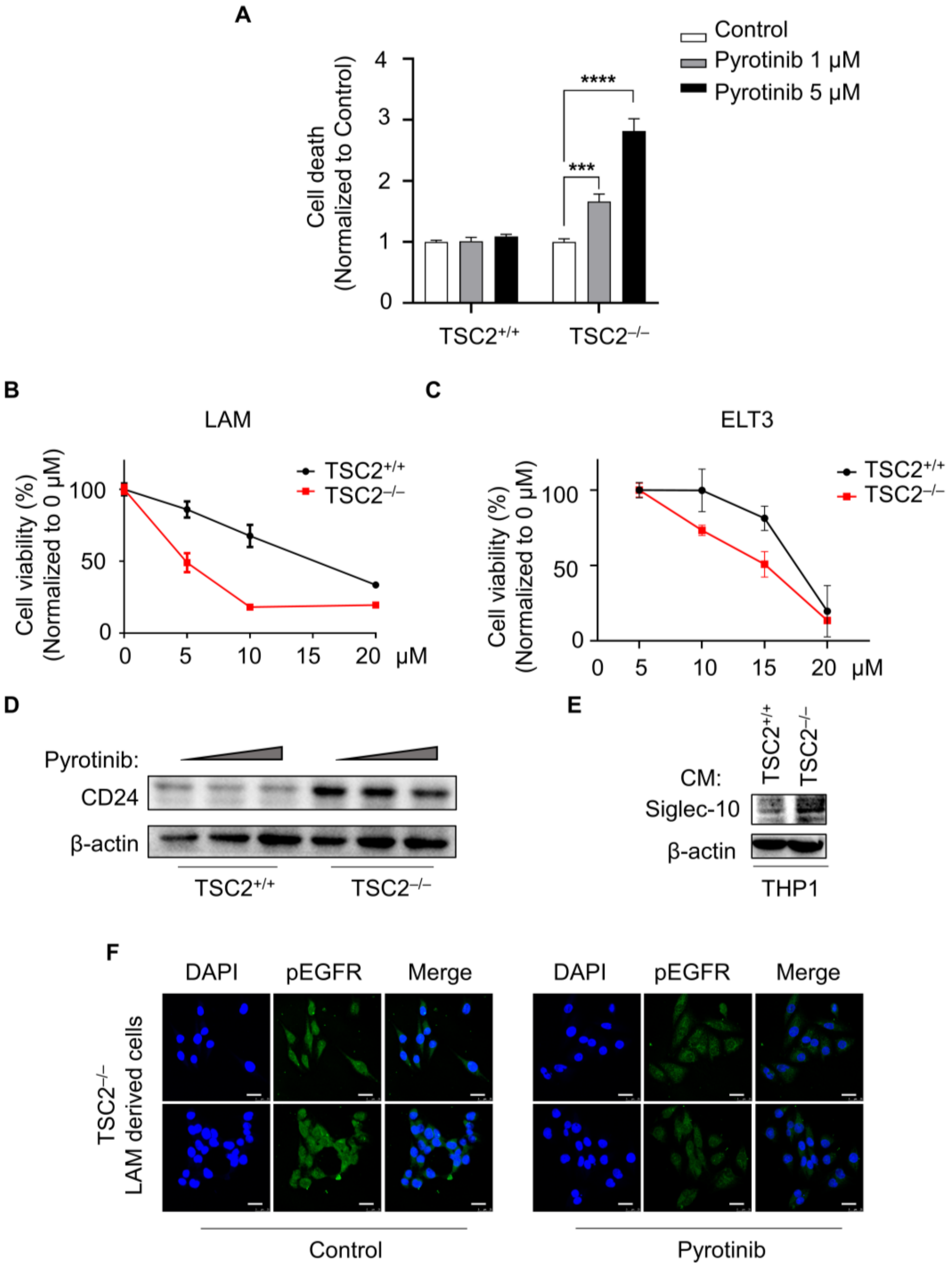


**Figure S1. Targeting efficiency of Pyrotinib in MEF/LAM/ELT3-derived cells. A**, MEF derived cells treated with or without pyrotinib (1 μM or 5 μM) for 48 h. Cell death was determined with the PI assay ($n$=5). ***$P < 0.001$, control versus pyrotinib (1 μM). ****$P < 0.001$, control versus pyrotinib (5 μM), Student $t$ test. **B and C**, LAM derived cells (**B**) and ELT3 derived cells (**C**) were treated with different concentration of pyrotinib for 48 h, and cell viability were detected by CCK8 assay, and IC50 of LAM derived TSC2-deficient cells was 4.404 μM, as while as 3.338 μM in ELT3 derived TSC2-deficient cells. **D**, Immunoblot analysis of CD24 in LAM derived cells treated with or without pyrotinib (1 μM or 5 μM). **E**, Macrophages of THP1 was co-incubated with culture supernatant of LAM with or without TSC2-deficient cells for 6 h, and THP1 cells were lysed, and siglec-10 was detected by immunoblot. **F**,

Localization and expression of pEGFR detected by immunofluorescence staining in TSC2-deficient LAM derived cells treated with or without pyrotinib (5 μM) (magnification 630×, bar = 25 μm).

| Gene Symbol | $TSC2^{+/+}$ | $TSC2^{-/-}$ | Log2Fold-Change ($TSC2^{-/-}$ vs. $TSC2^{+/+}$) | Pvalue | FDR |
|---|---|---|---|---|---|
| **Cd24a** | **0.2118336** | **5.5011925** | **5.2893589** | **5.00E-05** | **0.0004** |
| Cd1d1 | 1.7205636 | 0.6158119 | -1.104752 | 0.0316 | 0.12835 |
| Cd81 | 8.3645793 | 6.7701456 | -1.594434 | 5.00E-05 | 0.0004 |
| Cd300lb | 2.822726 | 0.0548058 | -2.76792 | 0.0424 | 0.162439 |
| Cd200 | 3.8694135 | 0.802024 | -3.067389 | 5.00E-05 | 0.0004 |
| H2-K1 | 0.8743655 | 1.8896489 | 1.0152835 | 0.00615 | 0.0322113 |
| Ildr2 | 0.018636 | 2.5172616 | 2.4986256 | 0.04235 | 0.162317 |
| Cd1d1 | 1.7205636 | 0.6158119 | -1.104752 | 0.0316 | 0.12835 |
| Il6st | 4.0059483 | 2.1793718 | -1.826577 | 0.0007 | 0.0046575 |
| Igsf10 | 1.0435959 | 0.0079094 | -1.035687 | 0.00705 | 0.0362655 |
| Pdgfra | 2.53234 | 1.1211969 | -1.411143 | 0.0002 | 0.0014727 |
| Tapbp | 4.935779 | 6.4111536 | 1.4753746 | 5.00E-05 | 0.0004 |
| Mr1 | 3.4339084 | 0.3484316 | -3.085477 | 5.00E-05 | 0.0004 |
| Cxcl12 | 0.5216163 | 5.746977 | 5.2253606 | 5.00E-05 | 0.0004 |
| Ccl20 | 0.5982164 | 4.25587 | 3.6576536 | 0.00295 | 0.0169383 |
| Bmp6 | 0.0982659 | 2.5554994 | 2.4572335 | 5.00E-05 | 0.0004 |

**Figure S2. Table of analysis of RNA-seq results in MEF cells.** The RNA-seq results was obtained from reference 13 (PMID: 34853810).

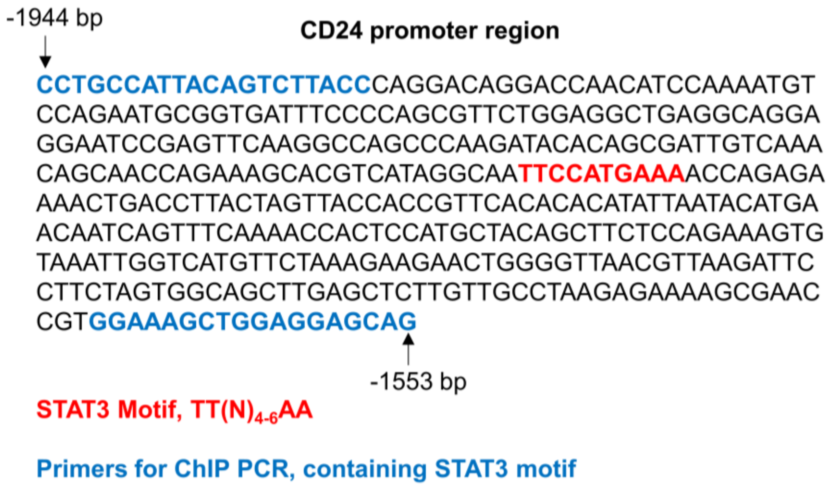


**Figure S3. ChIP PCR of CD24 promoter region designed for this study.** The full length of construct contained one important STAT3 binding motifs (-1778/-1769 bp), and primers for ChIP PCR sequence was designed by forward sequence: CCTGCCATTACAGTCTTACC and revised sequence: CTGCTCCTCCAGCTTTCC, including the promoter region of -1944/-1553

bp.

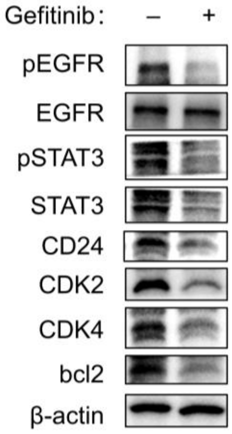


**Figure S4. Gefitinib used to validate the EGFR/STAT3/CD24 signaling.** Immunoblot analysis of MEF derived cells treated with or without Gefitinib (50 μM).

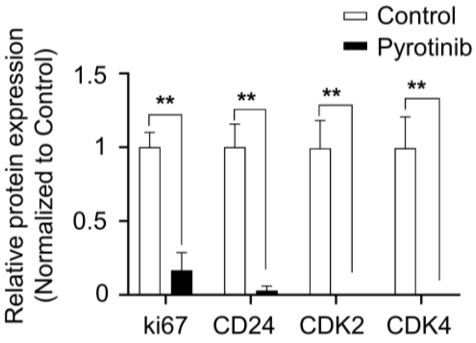


**Figure S5. Semi-quantified the proteins in Figure 5E.**
